# Does the Multistage 20-m Shuttle Run Test Accurately Predict VO_2max_ in NCAA Division I Women Collegiate Field Hockey Athletes?

**DOI:** 10.3390/sports9060075

**Published:** 2021-05-26

**Authors:** Meghan K. Magee, Jason B. White, Justin J. Merrigan, Margaret T. Jones

**Affiliations:** 1 Kinesiology, George Mason University, Manassas, VA 20110, USA; mmagee2@gmu.edu; 2 Frank Pettrone Center for Sports Performance, George Mason University, Fairfax, VA 22030, USA; whitej4@ohio.edu (J.B.W.); justin.merrigan@hsc.wvu.edu (J.J.M.); 3 Exercise Physiology, Ohio University, Athens, OH 45701, USA; 4 Human Performance Innovation Center, Rockefeller Neuroscience Institute, West Virginia University, Morgantown, WV 26506, USA; 5 Sport, Recreation, and Tourism Management, George Mason University, Fairfax, VA 22030, USA

**Keywords:** aerobic power, women athletes, Beep test, cardiovascular fitness, oxygen consumption

## Abstract

Laboratory assessments of maximal oxygen uptake (VO_2max_) are considered the “gold standard” for ascertaining cardiovascular fitness, but they are not always practical for use in team sport settings. Therefore, the purpose of the current study was to compare the criterion assessment of VO_2max_ on a treadmill to the progressive, multistage 20-m shuttle run test (i.e., Beep test), and to determine the predictability of 6 previously established Beep test predictive equations (i.e., Chatterjee, Flouris, Leger, Leger and Gadoury, Ramsbottom, St. Clair-Gibson). Collegiate women field hockey athletes (n = 65, mean±SD: age 19.6 ± 1.2 years; weight 64.7 ± 6.1 kg) completed criterion VO_2max_ (mean ± SD: 46.4 ± 4.6 mL**·**kg^−1^**·**min^−1^) and Beep tests to volitional fatigue. According to Bland–Altman and Ordinary Least Products Regressions, the Ramsbottom (46.5 ± 4.2 mL**·**kg^−1^**·**min^−1^) and Flouris (46.3 ± 3.8 mL**·**kg^−1^**·**min^−1^) equations were considered valid predictions of criterion measured VO_2max_ (46.4 ± 4.6). The Chatterjee, Leger, Leger and Gadoury, and St. Clair-Gibson equations overestimated VO_2max_, and are not recommended for use with women collegiate field hockey athletes. The Ramsbottom and Flouris estimates of VO_2max_ from 20-m shuttle performances may be used in this population. For accurate estimates of VO_2max_, the clientele’s age, fitness level, and training history should be considered when selecting equations.

## 1. Introduction

Field hockey is a field-based, intermittent, team sport comprised of short bouts of high intensity sprints and longer bouts of walking and jogging [1]. The aerobic energy system is predominant during collegiate women’s field hockey, as games consist of two 35-min halves with high-intensity movements comprising approximately 20% of that duration [2]. Further, according to time motion analysis, 95% of men’s field hockey competitions consist of low intensity tasks (e.g., walking, jogging, standing), while 5% of the competition is comprised of high intensity tasks (e.g., sprinting) [3]. Due to the evidence that field hockey is predominately an aerobic sport [3,4,5], testing for aerobic capacity is imperative in understanding the athletes’ preparedness for competition and for modification of training programs. 

Aerobic capacity can be validly and reliably assessed through measurement of inspired and expired gas exchange during a maximal graded exercise test [6]. The maximal oxygen consumption (VO_2max_) refers to the highest physiological value attainable, as indicated by a plateau in maximal oxygen consumption during exercise. However, VO_2peak_, the highest value obtained during exercise, should not be used interchangeably with VO_2max_. VO_2peak_ is more indicative of exercise tolerance than the maximal ability to transport and utilize oxygen, since individuals may select to discontinue exercise prior to achieving VO_2max_ [7,8,9,10]. Nonetheless, the aforementioned assessment of aerobic capacity requires expensive laboratory housed equipment and highly trained personnel, which are not always accessible or feasible for testing multiple athletes in team settings. Therefore, alternative, field based, methods for predicting VO_2max_ have been developed. A common and reliable test used to predict aerobic capacity is the progressive, multistage 20-m shuttle-run test or “Beep” test, in which the athlete runs 20-m laps at increasingly faster speeds until volitional fatigue [11,12,13]. Although VO_2max_ of men and women within the general population has proven to be correlated with the final shuttle speed during the Beep test [11,14,15], the ability of 20-m shuttle run tests to accurately predict VO_2max_ outcomes in collegiate athletes remains limited. 

In order to estimate VO_2max_ from Beep test performance, several equations have been developed that use the speed of the final stage achieved during the Beep test [15,16,17,18,19,20]. Equations have been validated within the general population [16,17,19], recreationally active men and women [15], children ages 8–19 years [20], and men squash and distance athletes [18]. However, there are differences in the aerobic capacity of athletes depending upon the sport in question, which may affect the validity of VO_2max_ equations from the Beep test in certain populations. Further, despite previously reported similarities in aerobic capacity between elite men and women field hockey athletes and endurance athletes [4], prediction of VO_2max_ can be dependent upon sex [21]. Thus, further investigation of the accuracy of several VO_2max_ prediction equations is warranted in various athletic settings, such as women field hockey athletes, to provide practitioners with appropriate equations for their population. This is critical for reliably providing VO_2max_ predictions, to reduce the chance of error in training prescriptions from overestimations or underestimations of VO_2max_. To assess true agreement between the predicted and criterion measured values, Bland–Altman and Ordinary Least Products Regressions are commonly conducted statistical procedures [22,23]. However, commonly reported statistics include correlational and linear regression analyses or t-tests, which assess the relationship or mean group differences between the assessments, not agreement. Therefore, this brings into question prior reporting on the accuracy of several 20-m shuttle VO_2max_ prediction equations. Thus, the aim of the current study was to determine the criterion validity of Beep test algorithms for predicting VO_2max_ in a sample of National Collegiate Athletic Association Division I women field hockey athletes.

## 2. Materials and Methods

### 2.1. Experimental Design

In a randomized order, field hockey athletes performed a laboratory-based maximal graded treadmill assessment and a field test of aerobic capacity at the same time of day on two days separated by 48 h. The laboratory assessment consisted of a continuous, incremental running protocol on a motorized treadmill. The field test was the progressive, multistage 20-m shuttle run test, also known as the Beep test. All athletes performed both assessments to volitional fatigue. Testing took place during the off-season. All athletes were familiar with treadmill running and the Beep test. 

### 2.2. Subjects

Sixty-five National Collegiate Athletic Association Division I women field hockey athletes (age, 20 ± 1 years; body mass, 64.7 ± 6.1 kg; body fat, 24.5 ± 5.5%; final Beep stage velocity, 12.7 ± 0.6 m∙s^−1^) participated in the current study. All athletes were under the direction of a certified strength and conditioning coach (NSCA-CSCS) and were following a similar training regimen. Athletes were instructed to refrain from exercise, alcohol, and supplementation 24 h prior to testing. Additionally, athletes were instructed to refrain from food and drink two hours prior to testing. All athletes completed a medical history form and were cleared for intercollegiate athletic participation. 

### 2.3. Criterion Measure of Maximum Oxygen Uptake

Laboratory assessment of VO_2max_ was conducted via a maximal graded treadmill (Marquette 1900, Milwaukee, WI, USA) protocol with 1-min stages. The protocol began at 3.0 mph and increased speed to 5.0 mph after one min. At the third min, speed was increased by 1.0 mph per min until speed was 8.0 mph. At min 6 and beyond, speed was held constant at 8.5 mph and only grade was increased by 1% per min. Heart rate was monitored continuously via heart rate monitor and watch (Polar Electro, Kempele, Finland), and the highest heart rate reached during each min was recorded. Oxygen consumption (VO_2_) was attained via expired gas analysis (VO2000 Metabolic System, MedGraphics, St. Paul, MN, USA), which was calibrated before each test according to manufacturer guidelines. The VO_2max_ corresponded to the highest VO_2_ reached prior to volitional fatigue. Achievement of VO_2max_ was dependent upon participants meeting three of the following criteria: RPE greater than or equal to 18; respiratory exchange ratio of 1.1 or greater; a plateau in VO_2_ (<150 mL∙min^−1^) despite an increase in workload; a maximal attained heart rate within 10 beats per min of age predicted heart rate maximum (206.9 *−* 0.67 × age); and a venous blood lactate > 8 mM. 

### 2.4. Progressive, Multistage 20-m Shuttle Run Test

The protocol followed for the progressive, multistage 20-m shuttle run (Beep) test was a commonly used modification [13,15,24,25] of the original developed by Leger and Lambert [11]. The Beep test was performed on an indoor basketball court with wooden floors and required athletes to run back and forth (“shuttle”) between two cones separated by 20 m. The initial speed was 2.22 m·s^−1^ for one min. At the end of the first min, the speed increased to 2.5 m·s^−1^ and progressively increased by 0.14 m·s^−1^ each min thereafter (Table 1). The speed was dictated by audible beeps from prerecorded audio, which was checked for accuracy prior to testing. Each min stage consisted of multiple “shuttles”, the number of which was dependent upon the stage speed. Athletes were instructed to keep running at the pace of the beeps for as long as possible, and they were grouped to provide a competitive environment. Once an athlete could no longer keep pace with the beeps (i.e., failed to complete two consecutive shuttles in time), the test was terminated. Estimated VO_2max_ was predicted from the maximal speed attained during the test via six previously developed prediction equations available in Table 2 [13,15,16,17,18,26]. 

### 2.5. Statistical Analysis

Data from criterion and Ramsbottom et al. [15] predicted VO_2max_ were normally distributed according to histogram plots and Shapiro–Wilks tests. However, due to the nature of the VO_2max_ predictions formulated from the final speed reached in the incremental treadmill test, these data were not normally distributed. Thus, these data do not meet the assumptions of standard parametric statistical assessments, such as correlations and t-tests. Nonparametric paired samples Wilcoxon Signed Rank tests and respective effect sizes were calculated to determine mean comparisons between predicted VO_2max_ from the criterion and Beep test equations.

Bland–Altman and Ordinary Least Products Regressions were run to determine the systematic and proportionate agreement between each predictive equation and the measured VO_2max_. The differences between the assessments were normally distributed and thus, assumptions of the Bland–Altman plots were met. Visualizations of the comparisons were created using Bland–Altman plots including 95% limits of agreement (mean bias ± [1.96 × SD of Differences]), their 95% confidence intervals, and a trendline of proportional bias and standard error. Bland–Altman plots and the respective statistics were created and assessed using BlandR package [27]. Since the interpretation of these plots has potential for error [23,28], ordinary least square products (Model II linear regression) analyses were performed to assess agreement between devices [29,30]. Systematic and proportional bias were noted when 95% confidence interval of the intercept did not include “0” and “1.0”, respectively. If the predicted VO_2max_ of an assessment resulted in systematic or proportionate bias, then this method should not be used interchangeably with criterion measured VO_2max_. All statistical procedures were conducted using R, version 3.6.2 (R Core Team, Vienna, Austria; https://www.R-project.org). The intercept, slope, and 95% confidence intervals of the Ordinary Least Products Regression analyses were calculated using the “sma” function within the “smatr” package [31]. For all statistical tests, alpha level was *p* < 0.05.

## 3. Results

Leger et al. [20], Leger and Gadoury [16], and St. Clair-Gibson et al. [18] predicted VO_2max_ values were significantly different, with large overestimations, from the criterion VO_2max_ (Table 3). 

**Figure 1:**
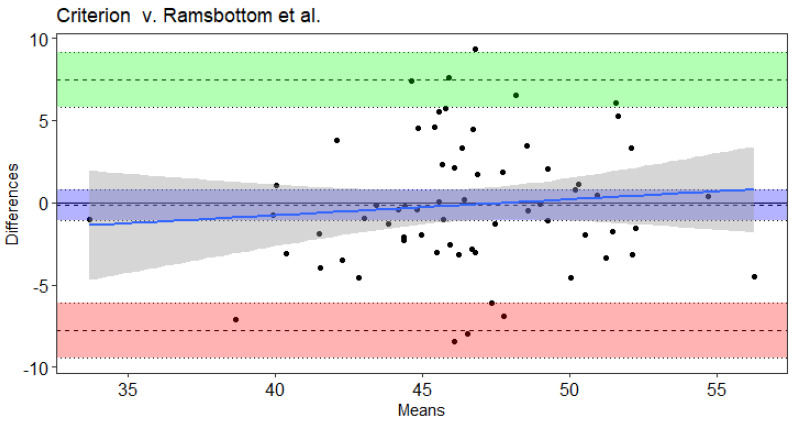
Bland–Altman plot of differences and means for criterion measured VO_2max_ from a maximal incremental treadmill test and the Ramsbottom et al. Beep test algorithm. The 3 dotted lines with blue shading represent mean bias, green shading represents upper 95% threshold, and red shading represents lower 95% threshold. The center line is either the mean difference or [1.96 × SD of Differences]), while the dotted lines above and below the center are the respective 95% confidence intervals. The solid blue line and the gray shading represent the trendline of proportional bias and the proportional standard error of the slope estimates, respectively.

**Figure 2:**
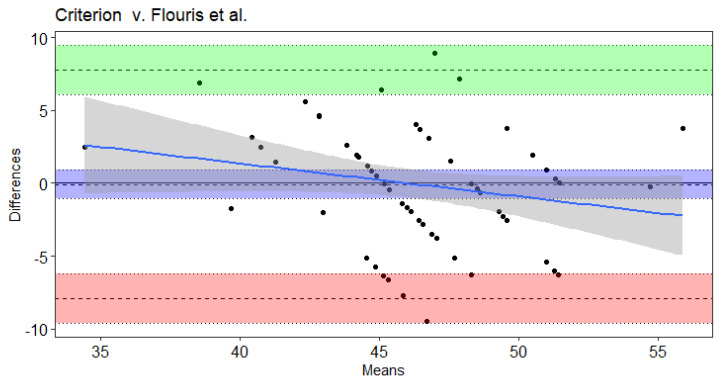
Bland–Altman plot of differences and means for criterion measured VO_2max_ from a maximal incremental treadmill test and the Flouris et al. Beep test algorithm. The 3 dotted lines with blue shading represent mean bias, green shading represents upper 95% threshold, and red shading represents lower 95% threshold. The center line is either the mean difference or [1.96 × SD of Differences]), while the dotted lines above and below the center are the respective 95% confidence intervals. The solid blue line and the gray shading represent the trendline of proportional bias and the proportional standard error of the slope estimates, respectively.

**Figure 3:**
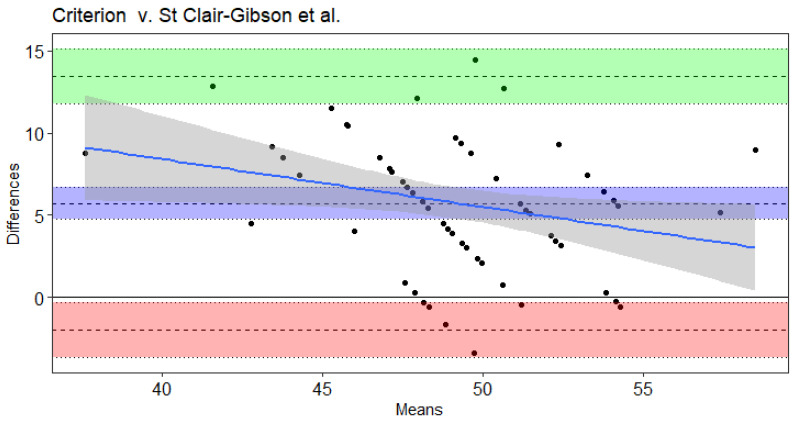
Bland–Altman plot of differences and means for criterion measured VO_2max_ from a maximal incremental treadmill test and the St. Clair Gibson et al. Beep test algorithm. The 3 dotted lines with blue shading represent mean bias, green shading represents upper 95% threshold, and red shading represents lower 95% threshold. The center line is either the mean difference or [1.96 × SD of Differences]), while the dotted lines above and below the center are the respective 95% confidence intervals. The solid blue line and the gray shading represent the trendline of proportional bias and the proportional standard error of the slope estimates, respectively.

**Figure 4:**
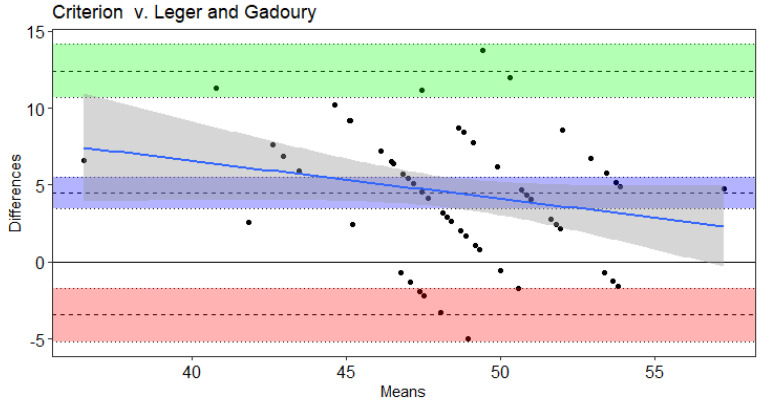
Bland–Altman plot of differences and means for criterion measured VO_2max_ from a maximal incremental treadmill test and the Leger and Gadoury Beep test algorithm. The 3 dotted lines with blue shading represent mean bias, green shading represents upper 95% threshold, and red shading represents lower 95% threshold. The center line is either the mean difference or [1.96 × SD of Differences]), while the dotted lines above and below the center are the respective 95% confidence intervals. The solid blue line and the gray shading represent the trendline of proportional bias and the proportional standard error of the slope estimates, respectively.

**Figure 5:**
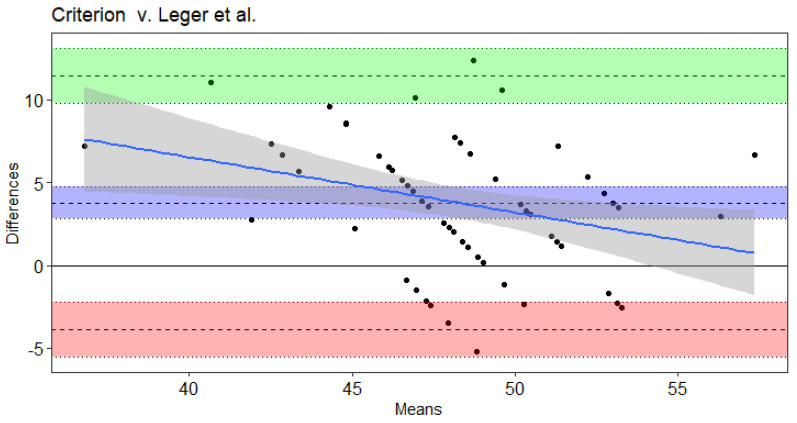
Bland–Altman plot of differences and means for criterion measured VO_2max_ from a maximal incremental treadmill test and the Leger et al. Beep test algorithm. The 3 dotted lines with blue shading represent mean bias, green shading represents upper 95% threshold, and red shading represents lower 95% threshold. The center line is either the mean difference or [1.96 × SD of Differences]), while the dotted lines above and below the center are the respective 95% confidence intervals. The solid blue line and the gray shading represent the trendline of proportional bias and the proportional standard error of the slope estimates, respectively.

**Figure 6:**
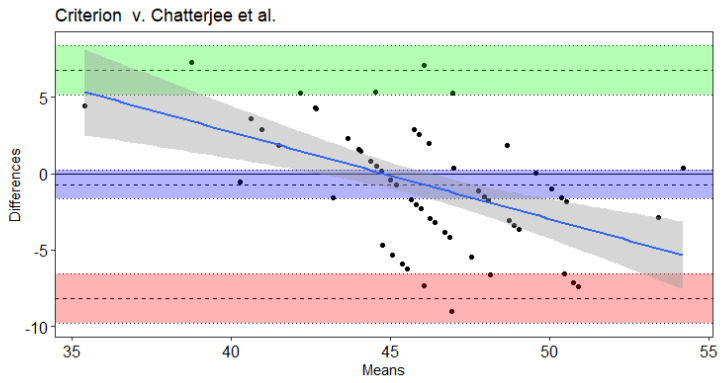
Bland–Altman plot of differences and means for criterion measured VO_2max_ from a maximal incremental treadmill test and the Chatterjee et al. Beep test algorithm. The 3 dotted lines with blue shading represent mean bias, green shading represents upper 95% threshold, and red shading represents lower 95% threshold. The center line is either the mean difference or [1.96 × SD of Differences]), while the dotted lines above and below the center are the respective 95% confidence intervals. The solid blue line and the gray shading represent the trendline of proportional bias and the proportional standard error of the slope estimates, respectively.

Results of the Bland–Altman and Least Products Regression analyses indicated true bias was not equal to 0 for Leger et al. [20], Leger and Gadoury [16], and St. Clair-Gibson et al. [18] (Table 4). The Ramsbottom et al. [15] (Figure 1) and Flouris et al. [19] (Figure 2) predicted VO_2max_ did not note bias according to Ordinary Least Products Regression or Bland–Altman procedures (Table 4). Systematic bias was indicated with wide limits of agreement for St. Clair-Gibson et al. [18] (Figure 3), Leger and Gadoury [16] (Figure 4), Leger et al. [20] (Figure 5). Least Products Regression analyses (Table 3) displayed systematic and proportional bias for Leger et al. [20] (Figure 5), Chatterjee et al. [17] (Figure 6), and St. Clair Gibson et al. (Figure 3) [18]. 

## 4. Discussion

The aim of the current study was to determine the criterion validity of Beep test algorithms for predicting VO_2max_ of National Collegiate Athletic Association Division I women field hockey athletes. The Leger, Leger and Gadoury, and St. Clair-Gibson equations largely overestimated VO_2max_. Yet, according to Least Products Regression and Bland–Altman analyses, the Leger, Leger and Gadoury, Chatterjee, and St. Clair-Gibson equations displayed systematic and proportionate bias when predicting VO_2max_. However, the Ramsbottom and Flouris equations were considered valid predictors of VO_2max_ in the current sample and thus, are considered appropriate equations for women field hockey athletes when compared to the other equations investigated in this study. Although the Ramsbottom and Flouris equations were statistically in agreement, the wide limits of agreement suggest each equation may have error in predictability up to 10 mL·kg^−1^·min^−1^. Thus, these equations should still be used with caution as comparisons at the individual level have displayed unacceptable error rates that would likely influence practical implications from the testing results.

The Ramsbottom et al. equation validly predicted VO_2max_ in the current study, but previously has underestimated VO_2max_ by 5.18–7.90 mL**·**kg^−1^**·**min^−1^ in male adolescent basketball players [32]. The underestimations in the aforementioned study may be a result of the higher average VO_2max_ of the male basketball players (55.45 ± 4.98 mL**·**kg^−1^**·**min^−1^). According to the current study, the Flouris et al. equation of the Beep test was also a valid predictor of VO_2max_. However, previous findings indicated the Flouris et al. equation to significantly underestimate laboratory VO_2max_ in women soccer athletes by 2.4 mL**·**kg^−1^**·**min^−1^ [13]. In each of the aforementioned studies, the protocol of the laboratory VO_2max_ tests were different from the current study, which may yield different results. Though the Flouris equation was validated in men, it is important to note that the VO_2max_ achieved by their sample was 46.9 ± 5.7 mL**·**kg^−1^**·**min^−1^, which is similar to that of the present study [19]. 

The Leger and Gadoury equation, developed in recreationally active adult men and women [16], overestimated VO_2max_ by ~4.5 mL·kg^−1^·min^−1^ in the current sample of women field hockey athletes. Previous literature has reported this equation to be valid in collegiate women soccer athletes [13]. Despite both of these athletic populations including women athletes in high aerobic demanding sports with similar VO_2max_ performances, differences between the criterion treadmill test of the Green et al. [13] study (using Leger and Gadoury equation) and current study may have resulted in discrepancies in the findings. Yet, the ~5.7 mL·kg^−1^·min^−1^ overestimation of the predicted VO_2max_ from the St. Clair-Gibson et al. equation is likely due to its development in men squash and endurance running athletes. The men squash players (63.4 ± 6.1 mL·kg^−1^·min^−1^) and endurance runners (69.6 ± 4.2 mL·kg^−1^·min^−1^) attained greater VO_2max_ values than women field hockey players in the current study (46.4 ± 4.6 mL·kg^−1^·min^−1^). However, the overestimations of the St. Clair-Gibson equation seemed to be greater at lower VO_2max_ levels, according to the proportional bias of Bland–Altman analyses.

St. Clair-Gibson et al. investigated the relationship between estimation of VO_2max_ and laboratory VO_2max_ in men athletes participating in squash (n = 10) and endurance running (n = 10), and reported the Ramsbottom equation to significantly underpredict VO_2max_ in endurance runners [18]. The cyclical nature of endurance running may render a 20-m shuttle assessment unsuitable for this population as endurance running does not involve a high volume of intermittent bursts of anaerobic effort nor frequent changes of direction. Additionally, previous literature has reported that predicted VO_2max_ determined from the Beep test requires significant contribution from anaerobic metabolism because of the need to slow down and accelerate every 20 m [13,33,34]. There was no difference between measured and predicted VO_2max_ values with squash athletes, which was attributed, in part, to the squash athletes’ skill in making calculated movements during intermittent exercise [35].

Contrary to current findings, the Leger et al. and Leger and Gandoury equations have resulted in accurate estimations of VO_2max_ in men Air Force cadets with a VO_2max_ of 56.8 ± 4.3 mL**·**kg^−1^**·**min^−1^ [12]. Of note, the Leger and Gandoury equation was developed in 53 males and 24 females aged 19–47 years with varying levels of aerobic fitness [16] while the Leger et al. equation was developed in a sample of children ages 8–19 years. Similar to the present study, Green et al. found the Leger and Gandoury equation to overestimate VO_2max_ by 5.4 mL**·**kg^−1^**·**min^−1^ in women soccer athletes [13]. Fitness level is another important characteristic to consider when predicting VO_2max_ from selected equations. The Chatterjee estimation equation was developed with college-aged women attending university in India [17]. Although the current study involved collegiate women subjects of similar age, they were trained, competitive athletes and of a higher fitness level than the untrained women in the Chatterjee study who obtained a VO_2max_ of 32.8 ± 2.9 mL**·**kg^−1^**·**min^−1^. This is evident within the results of the current study as the Chatterjee equation becomes increasingly inaccurate at greater VO_2max_ values. Findings from the current study in combination with previous literature highlight the importance of accounting for sex, sport, fitness level, and age when assessing aerobic fitness.

There are other necessary considerations, such as the individual’s age, fitness level, training history, and criterion measured VO_2max_ when analyzing the validity of estimation equations and comparing to prior literature. For example, Bland–Altman plots displayed the influence of an individual’s VO_2max_ on the accuracy of 20-m shuttle equations to estimate VO_2max_. Thus, discrepancies between the current and prior findings are likely attributed to differences in fitness level (VO_2max_). Discrepancies in findings may also be attributed to differences in statistical analyses used to test the validity of the equations. Although common in prior literature, use of correlation analyses or group mean comparisons to determine agreement are often inappropriate as they fail to provide valuable information for reliable comparisons [23]. For example, the Chatterjee equation was not significantly different from laboratory VO_2max_ according to group mean differences (i.e., Wilcoxon tests); however, according to Ordinary Least Products Regressions, the Chatterjee equation demonstrated systematic bias (underestimations) and proportional bias (changes in accuracy across VO_2max_ values). Thus, comparisons of average values between the criterion and equation estimated VO_2max_ are likely not accurate assessments of mean agreement. Instead, analyses such as Ordinary Least Products Regression and Bland–Altman analyses, that consider the mean and spread of agreement at the individual level should be conducted. 

## 5. Conclusions

The Ramsbottom and Flouris equations applied to 20-m shuttle run tests demonstrated valid estimations of VO_2max_ compared to criterion metabolic cart measures during maximal graded treadmill assessments. Thus, these equations may be a valid assessment of maximal oxygen consumption abilities of women field hockey athletes, when laboratory testing of VO_2max_ is unavailable. However, the equations should still be used with caution as the wide limits of agreement between the criterion and equations suggest high error rates at the individual level. The findings from the current study stress the importance of using caution when estimating VO_2max_ from prediction equations as results were inconsistent. In order to select the appropriate prediction equation, it is recommended that coaches and practitioners make themselves aware of the population involved in the equation’s development. Overestimations and underestimations of aerobic capacity may be detrimental for program design. This may lead to overtraining or deconditioning in athletes if the right estimation is not completed. For example, overestimations and underestimations of VO_2max_ would result in prescribed training intensities at VO_2max_ percentages higher or lower than an athlete should be training. Thus, intended adaptations may not persist due to prolonged training above or below necessary intensities. Practitioners should select an estimation equation that has been validated in a population as similar to their athletes and sport as possible. In order to achieve this, further research directed at the investigation of such equations in men and women athletes from different sports and training backgrounds is recommended.

## Figures and Tables

**Table 1:** Protocol for progressive, multistage 20-m shuttle run (Beep) test.

	Number of Shuttles	Speed (m·s^−1^)	Speed (km·h^−1^)
Stage 1	7	2.22	8
Stage 2	8	2.5	9
Stage 3	8	2.64	9.5
Stage 4	9	2.78	10
Stage 5	9	2.92	10.5
Stage 6	10	3.06	11
Stage 7	10	3.2	11.5
Stage 8	11	3.34	12
Stage 9	11	3.48	12.5
Stage 10	11	3.62	13
Stage 11	12	3.76	13.5
Stage 12	12	3.9	14
Stage 13	13	4.04	14.5
Stage 14	13	4.18	15
Stage 15	13	4.32	15.5
Stage 16	14	4.46	16
Stage 17	14	4.6	16.5
Stage 18	15	4.74	17
Stage 19	15	4.88	17.5
Stage 20	16	5.02	18
Stage 21	16	5.16	18.5

**Table 2:** The 20-m shuttle run prediction equation.

Reference	Equation
Ramsbottom et al. [15]	Table III
Leger et al. [20]	−24.4 + 6.0 × MAS
Leger and Gadoury [16]	(MAS × 6.592) − 32.678
Chatterjee et al. [17]	−14.956 + 4.78 × MAS
St. Clair-Gibson et al. [18]	6 × MAS − 24
Flouris et al. [19]	(MAS × 6.65 − 35.8) × 0.95 + 0.182

MAS, maximal aerobic speed.

**Table 3:** Mean comparisons of maximal oxygen uptake (VO_2max_) measurements.

Measurement	VO_2max_ (mL·kg^−1^·min^−1^)	*p*-Value	Effect Size	Effect Magnitude
Criterion	46.4 ± 4.6	--	--	--
Ramsbottom et al. [15]	46.5 ± 4.2	0.563	0.07	small
Leger et al. [20]	50.2 ± 3.5	<0.001	0.71	large
Leger and Gadoury [16]	50.9 ± 4.0	<0.001	0.77	large
Chatterjee et al. [17]	45.7 ± 2.9	0.185	0.16	small
St. Clair Gibson et al. [18]	52.1 ± 3.6	<0.001	0.82	large
Flouris et al. [19]	46.3 ± 3.8	0.932	0.01	small

Values are represented as mean ± standard deviation. p-values from Wilcoxon Signed Ranked tests and respective Rank Biserial effect sizes and magnitudes are displayed for comparison of Beep test calculations to criterion measures of VO_2max_.

**Table 4:** Least Products Regression and BlandR results for agreement between VO_2max_ predictions (df = 64).

VO_2max_ Equation	R^2^	Intercept (CI_95%_)	Slope (CI_95%_)	BlandR Stats
Ramsbottom et al. [15]	0.367 *	−3.925 (−14.051, 6.201)	1.081 (0.886, 1.319)	t = −0.303; *p* = 0.763
Leger et al. [20]	0.308 *	**−18.816 (−32.513, −5.120)**	**1.300 (1.056, 1.600)**	**t = 7.811; *p* < 0.001**
Leger and Gadoury [16]	0.308 *	−11.861 (−24.106, 0.384)	1.143 (0.929, 1.408)	**t = 9.059; *p* < 0.001**
Chatterjee et al. [17]	0.308 *	**−25.643 (−40.765, −10.520)**	**1.577 (1.281, 1.941)**	t = −1.508; *p* = 0.136
St. Clair-Gibson et al. [18]	0.308 *	**−19.076 (−32.827, −5.326)**	**1.256 (1.020, 1.547)**	**t = 11.714; *p* < 0.001**
Flouris et al. [19]	0.308 *	−8.865 (−20.485, 2.754)	1.193 (0.969, 1.469)	t = −0.162; *p* = 0.872

*, indicates significant R^2^. Bolded values indicate systematic bias (intercept CI_95%_ does not cross 0) and proportional bias (slope CI_95%_ does not cross 1).

## Data Availability

Data sharing is not applicable to this article.
